# Apoptosis in Porcine Pluripotent Cells: From ICM to iPSCs

**DOI:** 10.3390/ijms17091533

**Published:** 2016-09-12

**Authors:** Eunhye Kim, Sang-Hwan Hyun

**Affiliations:** 1 Institute for Stem Cell & Regenerative Medicine (ISCRM), Chungbuk National University, Cheongju 28644, Chungbuk, Korea; iwsleh@nate.com; 2 Laboratory of Veterinary Embryology and Biotechnology(VETEMBIO), Veterinary Medical Center and Collage of Veterinary Medicine, Chungbuk National University, Cheongju 28644, Chungbuk, Korea

**Keywords:** apoptosis, embryo, embryonic stem cells, induced pluripotent stem cells, pigs

## Abstract

Pigs have great potential to provide preclinical models for human disease in translational research because of their similarities with humans. In this regard, porcine pluripotent cells, which are able to differentiate into cells of all three primary germ layers, might be a suitable animal model for further development of regenerative medicine. Here, we describe the current state of knowledge on apoptosis in pluripotent cells including inner cell mass (ICM), epiblast, embryonic stem cells (ESCs), and induced pluripotent stem cells (iPSCs). Information is focused on the apoptotic phenomenon in pluripotency, maintenance, and differentiation of pluripotent stem cells and reprogramming of somatic cells in pigs. Additionally, this review examines the multiple roles of apoptosis and summarizes recent progress in porcine pluripotent cells.

## 1. Introduction

Apoptosis is a distinct mode of cell death that was first described in the 1970s [1]. It is considered essential for embryogenesis, organ metamorphosis, and tissue homeostasis. During embryo development, both in vitro and in vivo, the apoptotic process affects many cellular responses to suboptimal conditions and stress [2]. Therefore, it seems to play an important role in mammalian reproduction and development.

Among mammals, pigs are regarded as one of the ideal large animal species used in biomedical research. It is apparent that they are appropriate xeno-transplantation sources and may serve as a model for the study of human disease [3,4]. Their anatomical, immunological, and physiological characteristics are more comparable to humans than rodents. Even in comparison to nonhuman primates, pigs also have several specific advantages including short gestation intervals (114 days), cost-effectiveness, and production of multiple offspring (up to 12 piglets).

**Figure 1:**
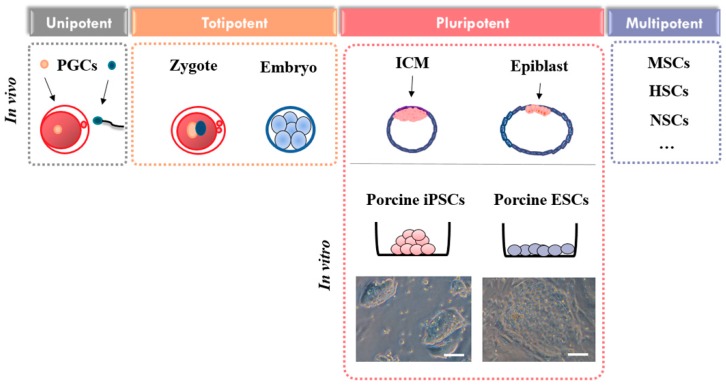
Stem cell state in vivo and in vitro. PGCs: Primordial germ cells, ICM: inner cell mass, iPSCs: induced pluripotent stem cells, ESCs: embryonic stem cells, MSCs: Mesenchymal stem cells, HSCs: Hematopoietic stem cells, NSCs: Neural stem cells. Scale bars = 50 µm.

Despite these advantages, obstacles still remain including limitations of using porcine pluripotent stem cells (PSCs) such as embryonic stem cells (ESCs) and induced pluripotent stem cells (iPSCs). Meanwhile, there is very significant ongoing research in the field of human and mouse PSCs. Here, we provide a brief overview of apoptosis and summarize some recently published reports focused on apoptotic events found in porcine pluripotent cells ranging from the inner cell mass in blastocysts and ESCs to iPSCs (Figure 1).

## 2. Blastocyst and Apoptosis

### 2.1. Preimplantation Embryonic Development

There is increasing evidence that apoptosis, including nuclear and cytoplasmic fragmentation, occurs during normal preimplantation of porcine embryo development in vivo and in vitro [5,6]. This incidence of apoptosis is a criterion for assessment of embryo quality and prediction of viability. Morphologically, the embryos shrink and become denser with fragmentation. However, it is not sufficient to appropriately assess the developmental capacity of an embryo following embryo transfer (ET) [7]. These apoptotic events in mammalian embryos have both beneficial and detrimental effects [8]. The removal of abnormal mutated cells by apoptosis plays an important protective role during embryo development. In contrast, if the ratio of apoptotic cells increases above the appropriate level, it could cause damage to normal blastomeres. Apoptotic events in normally developing embryos are not observed in the early stages of development prior to embryonic genome activation.

### 2.2. Inner Cell Mass (ICM) and Epiblast

After formation of the blastocoel within the porcine embryo, the blastomeres are partitioned into two distinct cell lineages: the inner cell mass (ICM) and the trophectoderm (TE). After implantation, the ICM differentiates into two cell lineages, the epiblast and the primitive endoderm, also called the hypoblast. The duration of porcine ICM and epiblast development is longer (approximately six to seven days) compared to that of mice and humans (one day for mice and three days for humans) [9]. The epiblast maintains pluripotency while the hypoblast develops into extraembryonic tissues in the early post-implantation stage. The frequency of apoptosis peaks during this stage and both cell lineages contain apoptotic cells [10]. Rauber′s layer, polar TE covering the epiblast, also undergoes apoptosis during this time. This layer becomes very thin and disappears during the extension of the epiblast. This is in accordance with earlier studies on porcine blastocysts [11]. The degeneration of Rauber′s layer in rabbits has been reported to exhibit apoptosis and subsequent phagocytosis by epiblast cells [12].

The final stage of the apoptotic process is commonly characterized by cell decay into apoptotic bodies. In pigs, apoptotic bodies were observed in embryonic disc D11 of the porcine blastocyst using transmission electron microscopy (TEM) [13]. Apoptotic blastomeres are phagocytosed by neighboring cells or extruded to the blastocoele or perivitelline space depending on the cell lineage [8]. The ICM cells tend to be adequately phagocytosed, whereas TE cells are easily extruded and undergo secondary necrosis. This differential susceptibility might be caused by the different environments in the ICM and TE.

## 3. Embryonic Stem Cells and Apoptosis

### 3.1. Porcine Embryonic Stem Cells (ESCs)

The ESCs are representative pluripotent cells. The porcine ESCs have especially received increased attention because of their potential to be used as powerful tools for genetic engineering aimed at improving transgenic animal production and as a possible source for cell replacement therapy [14,15]. However, no conclusive results have been obtained thus far and authentic porcine ESC lines have yet to be established. Almost all of the putative porcine ESCs generated previously have shown very similar characteristics as human ESCs, including colony morphology, signaling pathway, and inactivation status of the X chromosome (Table 1). In pigs, only a few groups reported the chimera contribution [16,17]. Although the capability of germline contribution in chimaeras is the most convincing criterion for authentic ESCs, there have been no reports on porcine ESCs that could contribute to the germ line until now.

From a perspective of apoptosis and ESCs, abnormal ESCs with mutations generated as a consequence of DNA damage might be removed from the self-renewal pluripotent population through apoptosis [18]. Indeed, there were a few reports on the apoptosis of porcine ESCs. While it will be difficult to make direct comparisons, some clues may be derived from the experiments reported in mouse and human ESCs to compare with the studies in porcine ESCs.

### 3.2. Metastable Pluripotent State and Mitochondrial Priming

The pluripotent state is not one single stage but rather is composed of at least two distinct types: the ICM type and epiblast type [19]. The ICM type is also referred as the naïve state (mouse ESCs) and the epiblast type is referred as the primed state (mouse epiblast stem cells (EpiSCs) and human ESCs). The porcine ESCs have acquired the typical characteristics of the primed (epiblast type) pluripotent state during derivation from the porcine ICM. Recently, the reverse conversion from primed to naïve state was induced in human ESCs by combining a change in culture conditions with an optimized small molecule inhibitor cocktail or with the overexpression of reprogramming factors [20,21]. These metastable pluripotent states of mammalian PSCs are relevant to apoptosis.

The ESCs, which give rise to all of the cell types in the body are known to be sensitive to DNA damage and undergo apoptosis. Both mouse and human ESCs have higher sensitivity to DNA damage compared with differentiated cells [22,23]. It was thought that ESCs, which might generate all of the tissues in the body, need to prevent damaged cells from compromising the genomic integrity of the population. The rapid apoptotic process of ESCs occurs to protect damaged cells from genomic instability if deleterious mutations are not repaired. Among the multiple pathways contributing to ESC sensitivity to DNA damage, the tumor suppressor protein p53 is a major mediator of response to double-strand breaks (DSBs) and single-stranded DNA (ssDNA) [24]. P53 acts as a transcription factor to upregulate multiple genes involved in apoptosis, such as BAX (Bcl-2-associated X protein), PUMA (p53-upregulated modulator of apoptosis), NOXA (meaning “damage”), APAF1 (apoptotic protease activating factor 1), and FAS (CD95). BAX is a major mediator of apoptosis and rapidly translocates to the mitochondria from the Golgi apparatus in its active conformation. Additionally, p53 can directly interact with cytoplasmic pro- and anti- apoptotic proteins [25].

**Figure 2:**
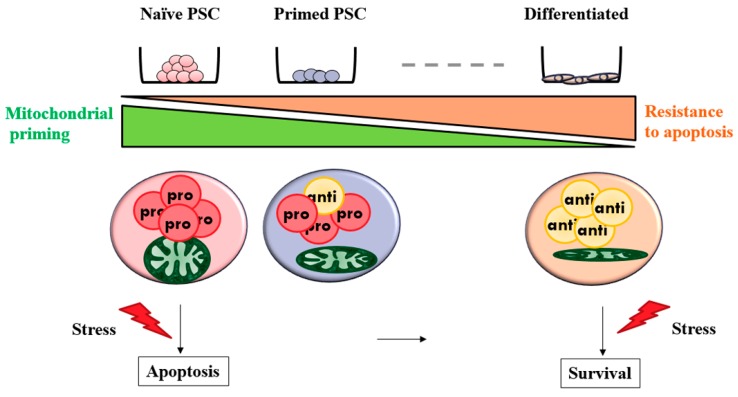
Mitochondrial priming and apoptosis in pluripotent stem cells. PSC; Pluripotent stem cells, Differentiated; differentiated cells, Pro; pro-apoptotic factors, Anti; anti-apoptotic factors.

However, p53 transcriptional activity is not a clear indicator for distinguishing ESCs from differentiated progeny cells. The sensitivity to apoptosis of ESCs is correlated with mitochondrial priming, also called an apoptotic threshold [26]. The ESCs are maintained close to the apoptotic threshold, which is determined by the balance between pro- and anti- apoptotic BCL2 family proteins, which are have been proposed to play a central role in regulating apoptosis, and measured via BCL2 homology domain 3 (BH3) profiling. Recently, according to Julia C. Liu et al., one of the potential mechanisms controlling the primed state of human ESCs might be the regulation of the balance of mitochondrial priming [27]. Additionally, mitochondrial morphology is regarded as a novel factor for determining pluripotent states [28]. Consistent with this, our recent work has also demonstrated that porcine ESCs derived from porcine clone blastocysts using iPSCs as donors (iPS-NT ESCs)—which have relatively more naïve pluripotent features—have a more immature form of the mitochondrial organelle shape with under-developed cristae than other origins regarded as the primed state [29]. This result suggested that a difference in mitochondrial dynamics is present not only between ESCs and differentiated cells but also between pluripotent stem cells of different pluripotent states (naïve vs. primed). The relationships between the mitochondrial dynamics, pluripotent states, and apoptosis are illustrated in Figure 2. The mitochondrial structures of primed PSCs such as porcine ESCs are more immature than differentiated cells, but more mature than naïve PSCs. The PSCs initiate apoptosis in response to a cellular stress such as DNA damage, whereas differentiated cells may survive under the same stress. This means that PSCs exist in a higher state of mitochondrial priming with an elevated ratio of pro-apoptotic to anti-apoptotic proteins present compared to their differentiated cell types.

Then, what is the cause of this relationship? It is possible that mitochondrial priming and apoptosis are correlated with cell cycle. The PSCs have a shortened cell cycle due to a shorter G1 phase compared to differentiated cells. This rapid transition of G1-S phase in PSCs might be caused by the more immature from of mitochondria, which is morphologically fragmented and metabolically inactive in comparison to the differentiated counterpart. We will discuss the details of differentiation and apoptosis in the following section.

### 3.3. Differentiation

The profiling of BH3 proteins confirmed that the higher mitochondrial priming of ESCs declined as the cells differentiate [26]. Dynamic differences of mitochondria caused by fission and fusion process might be coordinated with cellular processes such as cell metabolism and differentiation [30]. It has become obvious that murine ESCs express high levels of glycolytic enzyme and exhibit low mitochondrial oxygen consumption [31]. However, the switch of the energy production from glycolysis to mitochondrial respiration and oxidative phosphorylation was observed during differentiation. The differentiated cells have higher levels of mitochondrial mass and oxidative phosphorylation. This conversion of energy metabolism was also known to occur during osteogenic differentiation from human mesenchymal stem cells [32]. Our previous report confirmed that the porcine ESCs in a higher passage have much more initial signs of apoptosis than those in lower passages through TEM analysis [33]. As porcine ESCs are passaged under stress, some of them exhibit pyknosis, wrinkly nuclear envelopes, and numerous lysosomes associated with autophagic vacuoles. This might explain, at least in part, why the porcine ESCs are sensitive to DNA damage and quickly undergo apoptosis using glycolysis as a major source of energy.

The recent bulk of accumulating evidence has contributed to knowledge facilitating a better understanding of apoptosis in adult stem cells (ASCs) and terminally differentiated cells. In general, it was thought that ASCs are more resistant to cell death following damage compared to ESCs [34]. However, there are some variations in sensitivity to DNA damage among ASCs (reviewed in [27]). The hematopoietic stem cells (HSCs) or keratinocyte stem cells (KSCs) are more resistant, whereas candidate stem cells in the small intestinal crypt are more sensitive to DNA damage compared with their committed progenitors. Nevertheless, the dynamic balance of sensitivity and resistance to DNA damage among the self-renewal, differentiated, and stem cell populations of ASCs are not fully understood.

During differentiation, there are some changes in the mitochondrial function of cells. The mitochondrial alterations are involved with typical apoptotic features. In neural differentiation, for instance, these mitochondrial apoptotic events include a p53 mitochondrial translocation, cytochrome c release, and production of reactive oxygen species (ROS) [35]. When the differentiated cells are aged, they exhibit a slow proliferation rate, shortening of the telomeres, and upregulation of p53. These features trigger cellular senescence and apoptosis leading to cell death. In contrast, cancer cells did not enter this growth arrest state and proliferated indefinitely [36]. The intricate relationship between cancer and apoptosis is supported by the evidence that abnormal expression of some key apoptotic regulatory factors leads to cancer [37]. It is now clear that some oncogenic mutations disrupt apoptosis, leading to cancer initiation, progression, or metastasis. Oct-4, a POU homeobox transcription factor, was recently shown to be transcribed in cancer cells and might contribute to the survival of cancer cells [38], but its biological functions have remained unclear. Recently, overexpression of Oct-4 in cancer cells was reported to promote carcinogenesis, and inhibit cancer cell apoptosis [39]. In PSCs, Oct-4 expression appears to be required for maintenance of the undifferentiated pluripotent state, and it may play a protective role in stress-induced apoptosis. The Oct-4 knocked-down in murine ESCs was significantly increased in response to stresses compared with parental cells [40]. Considering the low apoptotic threshold described in the above section, it will be challenging and interesting to study this contradictory action of Oct-4 associated with apoptosis and pluripotency in PSCs including porcine ESCs.

### 3.4. Subculture

Porcine ESCs easily undergo apoptosis if they are dissociated into single cells for subculture [41]. They must be kept as cell clumps during passaging because of the hypersensitivity to apoptosis when dissociated. This is similar to conventional human ESCs [42]. However, this dissociation-induced apoptotic problem was solved by the inhibition of the Rho/ROCK (Rho-associated kinase) signaling pathway with ROCK inhibitors such as Y27632 [43]. A recent study revealed that a ROCK inhibitor permits the survival of dissociated single human ESCs in both adhesion and suspension cultures through inhibiting myosin II activity, major downstream effector of ROCK [44,45]. They found that the contractile force within a cell, induced by activation of myosin II, could be reduced by ROCK inhibitors associated with RHO-ROCK-MLC (myosin light chain) signaling. Furthering these studies, the ROCK-dependent apoptosis in porcine ESCs needs to be analyzed to solve the fragility problem upon dissociation, which is a large obstacle to developing porcine ESC manipulation techniques.

## 4. Reprogramming and Apoptosis

### 4.1. Porcine Induced Pluripotent Stem Cells (iPSCs)

**Figure 3:**
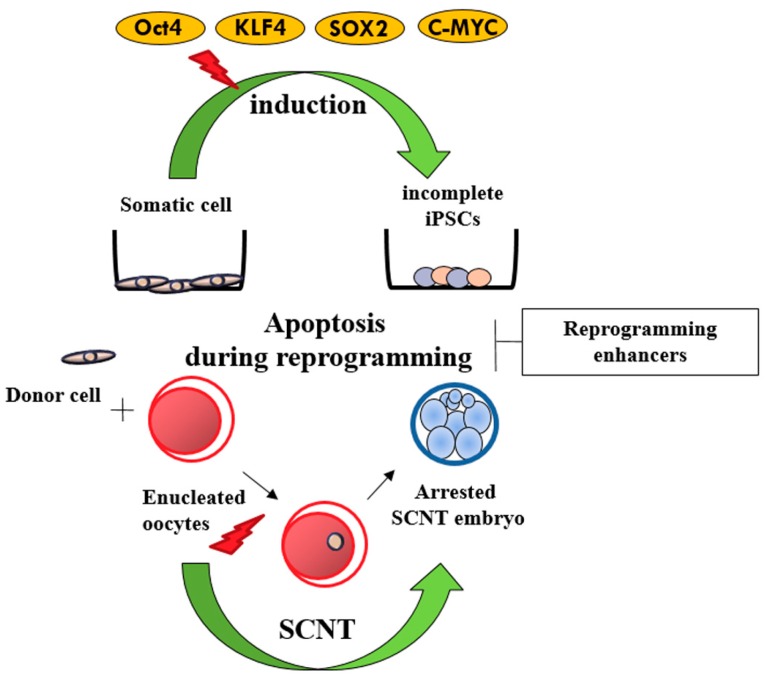
Apoptosis during reprogramming. iPSCs: induced pluripotent stem cells, SCTN: somatic cell nuclear transfer.

The improvement of cell reprogramming technology provided a new strategy for the generation of reprogrammed cells from differentiated cells called iPSCs. The first iPSCs were produced by reprogramming somatic cells in mice with four Yamanaka cocktail of stemness factors such as *POU5F1*, *SOX2*, *KLF4*, and cMYC (known as OSKM) using integrating retroviral vectors [46]. These iPSCs have normal karyotypes, express cell surface markers and genes that characterize ESCs, and showed the developmental capacity to differentiate into all three primary germ layers. Several studies have shown that during iPSC induction, apoptotic processes and cellular senescence occur in the first few days after overexpression of reprogramming factors [47]. The first porcine iPSCs were generated by Ezashi et al. [48], which have basic fibroblast growth factor (bFGF)-dependent signaling similar to epiblast type ESCs such as those seen in mouse EpiESCs or conventional human ESCs. As shown in Figure 3, the reprogramming of porcine cells, which is a stressful process, also activates this apoptotic phenomenon contributing to the low efficiency of iPSC generation. One study in porcine iPSCs reported that when the Lif-2i medium was added one day later, the transduced cells failed to expand and entered apoptosis during further culture [49].

A major mediator acting in the apoptotic pathway is the tumor suppressor protein, p53. The p53-induced intrinsic apoptotic pathway occurs during iPSCs generation. Reports in human and mouse iPSCs supported this by showing that knockdown of p53, or its target gene p21, improved the efficiency of iPSC generation, and thus suggested p53 is critically involved in limiting iPSC induction [50,51,52]. C-Myc, one of the Yamanaka factors, is able to activate the apoptotic pathway via the p53–p21 pathway. Although the absence of C-Myc reduced apoptotic rate to 1%–3% [53], the other three factors (OSK) also activate p53 as evidenced by the activation of two key apoptotic proteases, caspases 3 and 8, by Oct-4. Additionally, the Blasco group demonstrated that p53 has a crucial role in preventing the reprogramming of cells carrying various types of DNA damage, including short telomeres [51]. Telomere reprogramming during iPSC induction and passage of iPSCs increased telomere damage and shortening. The incomplete telomere reprogramming and maintenance are associated with active exogenous genes in porcine iPS cells [54]. Indeed, most porcine iPSCs displayed incomplete silencing of exogenes [55,56] unlike the complete silencing seen in mouse and human iPSCs [57,58].

The mouse-ESC-like porcine iPSCs can also be produced using culture-conditioned medium for the ground state such as 2i plus leukemia inhibitory factor (LIF) [56]. This ICM type of porcine iPSCs can generate chimeras with germline competence and produce cloned piglets [59,60]. These two types of porcine iPSCs (ICM type vs. Epiblast type) have different gene expression patterns and different signaling pathways for maintenance of stemness. Unlike epiblast-type porcine iPSCs and ESCs, ICM-like naïve porcine iPSCs are not vulnerable to dissociation and can be passaged using enzymatic methods. A recent study reports that porcine 6F iPSCs induced by Oct-4, Sox2, Klf4, c-Myc, T-Box 3 (Tbx3), and liver receptor homologue-1 (LRH1, also known as Nr5α2) could be single-cell passaged similar to mouse ESCs, and unlike porcine 4F iPSCs induced by the classical four factors, OSKM [61]. Notably, the two transcription factors Tbx3 and Nr5α2 might improve the viability of porcine iPSCs after dissociation into single cells by inhibiting the RHO-ROCK-MLC signaling pathway. This is in accordance with the earlier referred report in human ESCs [43].

MicroRNAs (miRNAs) are small non-coding transcripts of –22 nucleotide (nt) which are involved in multiple biological processes, including early mammalian development and stem cell maintenance and differentiation [62]. In mammalian primed pluripotent stem cells, some miRNAs including miR-20, miR-92, and miR-302 regulate the apoptotic threshold and survival through targeting the pro-apoptotic protein BIM [63]. The expression of the miR302/367 cluster especially allows the rapid and efficient reprograming of mouse and human somatic cells to an iPSC state without forced expression of exogenous transcription factors [64]. Moreover, recent findings suggest that porcine iPSCs generated by manipulating the culture conditions (treatment of LIF, bFGF, and bone morphogenetic protein (BMP4) with two inhibitors (CHIR99021 and SB431542)) exhibited intermediate pluripotent state showing mixed miRNA profiles of naïve and primed pluripotency [65]. The small RNA sequencing data showed the upregulation of the miR-302b/367 and miR-106a/363 clusters, and downregulation of let-7 family members and the miR-17/92 cluster. Altogether, this suggests that intensive study of non-coding RNAs such as miRNAs in porcine PSCs will provide a superior insight into the regulatory networks that underlie developmental processes, self-renewal, differentiation, and cellular reprogramming.

### 4.2. Somatic Cell Nuclear Transfer (SCNT)

Apoptosis has important roles in eliminating cells that are abnormal or detrimental. Several studies reported that the incidence of apoptosis is higher in embryos produced in vitro than those produced in vivo [7,66]. Among the in vitro produced embryos, SCNT embryos displayed a very high degree of apoptosis even compared to in vitro fertilized (IVF) embryos as reported in porcine experiments [67]. This might be caused by the fact that reprogramming occurs during nuclear transfer of somatic cells, as in the iPSC generation process. Apoptotic triggers to induce the expression of genes involved in cell death may act during reprogramming. As the success rate of SCNT remains low, researchers are trying to improve the developmental potential of reconstructed mammalian embryos. In this regard, one of the strategies for successful SCNT is reducing apoptosis to enhance the reprogramming efficiency by small molecules such as a histone deacetylase (HDAC) inhibitor, a ROS inhibitor and cytokines. For instance, a recent study revealed that trichostatin A (TSA), an inhibitor of HDAC, improved the developmental competence of cloned porcine embryos by improving epigenetic reprogramming [68]. The TSA-induced apoptosis during the initial embryonic stage allows abnormal cells to be eliminated, thus enhancing proliferation of healthy normal cells and improving quality. Elevated levels of ROS have been detected within arrested NT embryos. Porcine SCNT embryos treated with vitamin C, a common ROS inhibitor, showed a significantly higher blastocyst development rate than that of untreated SCNT embryos [69]. Our previous report also found that the addition of porcine granulocyte-macrophage colony-stimulating factor (GM-CSF) might modulate apoptosis in SCNT-derived blastocysts and increase the viability and developmental competence of cloned embryos [70]. Nevertheless, the comprehensive strategies for control of apoptotic events in porcine SCNT embryos during development in vitro have not been reported to date. However, it will be essential to identify apoptosis-related phenomena during cloned embryo development in vitro, which may provide insight into the relationship between the process of apoptotic cell death and the efficiency of porcine SCNT.

## 5. Concluding Remarks and Future Perspectives

The present review has been confined to serial apoptotic-related phenomena in porcine pluripotent cells, with an emphasis on late embryo development, metastable pluripotent states, hypersensitivity, differentiation, and reprogramming. It will be an interesting challenge to explore the interconnections between pluripotency, cell cycle, and mitochondrial dynamics in porcine PSCs. A better understanding of apoptosis in porcine pluripotent cells as well as their modulation would be expected to bring substantial benefit to researchers studying a wide range of biomedical and regenerative medicine. Remarkably, the study of apoptosis arising during derivation, maintenance, or differentiation of porcine ESCs and iPSCs may provide insights into technical improvements for generation of authentic porcine PSCs as a tool for disease modeling.

## Figures and Tables

**Table 1:** Characteristic comparison of mouse ESCs, human ESCs, and porcine ESCs.

Properties	Mouse ESCs	Human ESCs	Porcine ESCs
Colony morphology	Small rounded dome shaped	Monolayer epithelium	Monolayer epithelium
Signaling pathway	LIF/BMP dependent	FGF/Activin dependent	FGF/Activin dependent
X chromosome inactivation	XaXa	XaXi	XaXi
Apico-basal polarity	−	+	+
Surface marker	SSEA1	SSEA3/4, TRA-1-60, TRA-1-81	SSEA3/4, TRA-1-60, TRA-1-81
EB formation	+	+	+
Chimera contribution	+	N/A	+
Germline transmission	+	N/A	−

Embryonic stem cells (ESCs); Leukemia inhibitory factor (LIF); Bone morphogenetic protein (BMP); two active X chromosomes (XaXa); one inactivated X choromosome (XaXi); presence of the property (+); absence of the property (−); Embryoid body (EB).
